# Male breast cancer: common biomarkers, clinicopathology, and outcomes in the west of Iran

**DOI:** 10.1186/s43046-025-00294-y

**Published:** 2025-05-07

**Authors:** Ali Azizi, Nasrin Mansouri, Bayan Faridi, Mazaher Ramezani

**Affiliations:** Kermanshah University of Medical Sciences, Kermanshah, Islamic Republic of Iran

**Keywords:** Male, Breast cancer, Prognostic factors, Survival

## Abstract

**Background:**

Male breast cancer is a rare disease that accounts for less than 1% of all cancers in men and less than 1% of all diagnosed breast cancers. We retrospectively evaluated clinicopathologic features, treatment options, and overall survival in male breast cancer cases over 10 years (2012–2021).

**Methods:**

In this descriptive-cross-sectional study, the men with a breast cancer patient information based on demographic characteristics, type of surgery performed, pathological characteristics of samples (including the type of tumor involving lymph nodes and its grade), distant metastasis, immunohistochemical markers as well as family history of cancer, number of chemotherapy and radiotherapy sessions, use of anabolic drugs, and patient survival after surgery were recorded in the designed checklist.

**Results:**

The results showed that the mean age of men with breast cancer was 56.14 ± 14.59. Invasive ductal carcinoma was diagnosed in 86.3% of patients. In addition, metastasis occurred in 23.5% of patients, and most metastases occurred in the liver and then in the bone marrow, respectively. The highest frequency was related to stage IIB, with a frequency of 29.4%. The overall survival rate of 1, 3, and 5 years for 51 cases was 96%, 91%, and 65%, respectively, with an average survival period of 96 months. There was a significant relationship between age, metastasis, and disease stage with the survival status of patients (*P* = 0.03).

**Conclusions:**

In the present study, old age, higher stage, and metastasis in male breast cancer were associated with unfavorable survival.

## Introduction

Breast cancer in males currently represents 0.5% of all cancer diagnoses among men annually and constitutes approximately 1% of all breast cancer cases in the USA [1]. Despite its rarity, the incidence of male breast cancer is increasing [2].

Men diagnosed with breast cancer have historically been underrepresented in clinical trials and population-based research, primarily due to the rarity of the condition. Currently, there are no reported outcomes from prospective national or international clinical studies that focus exclusively on male breast cancer patients. Given the limited availability of male-specific data, clinicians must rely on findings from studies that primarily involve female breast cancer patients to inform disease management strategies. Consequently, the treatment protocols for male breast cancer patients are largely analogous to those employed for postmenopausal women [3].

While breast cancer constitutes a relatively small proportion of all malignancies diagnosed in males, it is associated with one of the highest mortality rates. According to the American Cancer Society, in 2019, the incidence of testicular cancer diagnoses in men was more than three times that of breast cancer. Notably, over the preceding 5 years, a greater number of men succumbed to breast cancer compared to testicular cancer [4]. These findings underscore the necessity for ongoing research focused on male-specific breast cancer. Consequently, population-based studies may contribute to the development of treatment guidelines and recommendations by providing critical insights into these rare tumor types. Breast cancer is categorized into subtypes based on the expression of the estrogen receptor (ER), progesterone receptor (PR), and human epidermal growth factor receptor 2 (HER2) [5].

In the context of female breast cancer, substantial evidence underscores the importance of tumor subgroups in prognostic assessments, as well as the efficacy of chemotherapy, hormonal therapy, and targeted treatments. Conversely, male breast cancer is characterized by a limited body of evidence. Consequently, the majority of male patients are managed according to the treatment guidelines established for female patients. Due to the rarity of male breast cancer, the majority of retrospective studies have encompassed breast cancer diagnoses spanning several decades [1–5].

While this approach enhances the sample size, it is suboptimal as it fails to account for advancements in prognosis related to screening and treatment over time, as well as other prognostic factors that have only recently been incorporated into registry data, such as estrogen and progesterone receptor status, which may vary between male and female patients. Numerous studies have indicated that prognostic variables, epidemiological factors, and tumor behavior exhibit differences between men and women diagnosed with breast cancer [2, 6–8].

The identification of discrepancies in breast cancer survival outcomes may facilitate the development of interventions aimed at improving care for male breast cancer patients. However, existing research has yielded inconsistent findings. While certain studies indicate that males with breast cancer experience a poorer prognosis compared to females, other investigations suggest that men may have comparable or even superior outcomes [9–13].

This study aims to investigate the characteristics and outcomes of male breast cancer patients in western Iran from 2012 to 2021, given that, in contrast to female breast cancer, there have been a limited number of studies conducted on male breast cancer.

## Methods

This descriptive cross-sectional study focused on a population of men diagnosed with breast cancer who were referred to hospital over a 10-year period, from January 2012 to December 2021. Data were collected regarding various demographic characteristics, including age, education, region of residence, and occupation. Additionally, information pertaining to the type of surgical intervention performed, pathological characteristics of the samples (such as tumor type, lymph node involvement, and tumor grade), instances of distant metastasis post-surgery, immunohistochemical markers (including ER, PR, AR, P53, and Ki67), family history of cancer, the number of chemotherapy and radiotherapy sessions, use of anabolic drugs, and patient survival following surgery was systematically recorded using a designated checklist. Furthermore, patients whose cancer stages, as defined by the cancer staging manual, were inconsistent with specific site metastases, as well as those with incomplete follow-up information, unknown follow-up durations, or inaccurate follow-up timelines, were excluded from the study.

### Statistical analysis

Data Analysis was performed using SPSS statistical software (version 20). The means and standard deviations of the quantitative findings, as well as the frequency percentages of the qualitative results, were reported. To identify significant relationships, the *t*-test, chi-square test, and Mann–Whitney *U*-test were employed.

## Results

In this study, a total of 51 male patients diagnosed with breast cancer were referred to Imam Reza Hospital in Kermanshah, Iran, during the period from 2012 to 2021. The results indicate that the mean age of the male patients with breast cancer was 56.14 years (± 14.59), with a median age of 54 years, and an age range spanning from 32 to 100 years. A significant majority of the patients (76.5%) resided in urban areas, and self-employment was identified as the most prevalent occupation among the participants, accounting for 68.6% of the cases (Table 1).

**Table 1:** Demographic characteristics of breast cancer patients (*n* = 51)

Variable	Number (%)
**Age**	
< 50	20 (39.2)
51–64	19 (37.3)
> 64	12 (23.5)
**Education**	
Illiterate	12 (23.5)
Elementary	34 (66.7)
Guidance	3 (5.9)
Diploma	2 (3.9)
**Area of residence**	
Urban	39 (76.5)
Rural	12 (23.5)
**Occupation**	
Employee	9 (17.6)
Free	35 (68.6)
Unemployed	7 (13.7)

According to the data presented in Table 2, invasive ductal carcinoma was identified in 86.3% of the patients studied. Additionally, metastasis was observed in 23.5% of the patients, with the liver and bone marrow identified as the most prevalent sites of metastasis. The highest frequency of metastasis was associated with stage 2B, reported at 29.4%. The mean tumor size was measured at 2.75 ± 1.16 cm, with a range from 1 to 7 cm. Lymph node metastasis was noted in 76.47% of the patients; furthermore, 84.3% of these patients had fewer than two involved lymph nodes, while 15.7% had more than two. Additionally, 47.05% of the patients reported a family history of cancer, 76.6% underwent surgical mastectomy, and 25.5% received an excisional biopsy. Chemotherapy regimens including docetaxel, doxorubicin, and cyclophosphamide (TAC), as well as cyclophosphamide, epirubicin, and fluorouracil (CEF), were administered to 43 cases, representing 84.3% of the cohort.

**Table 2:** Pathological and clinical characteristics of breast cancer patients (*n* = 51)

Variable	Number (%)
**Tumor location**	
Right breast	23 (45.1)
Left breast	28 (54.9)
**Tumor type**	
In situ ductal carcinoma	44 (86.3)
Invasive ductal carcinoma + in situ	1 (2)
Invasive lobular carcinoma	3 (5.9)
In situ lobular carcinoma	1 (2)
Papillary carcinoma	1 (2)
In situ component	1 (2)
**In situ ductal carcinoma**	
Yes	17 (33.3)
No	34 (66.7)
**Metastasis (after surgery)**	
Yes	12 (23.5)
No	39 (76.5)
**Metastasis to organs**	
Liver	6 (50)
Bone marrow	5 (41.66)
Kidney	1 (8/33)
**Stage**	
IA	6 (11.8)
IB	1(2)
IIA	13 (25.5)
IIB	15 (29.4)
IIIA	3 (5.9)
IIIB	1 (2)
IV	12 (23.5)
**Type of surgery**	
Mastectomy	36 (76.6)
Excisional biopsy	13 (25.5)
Lumpectomy	1 (2)
Quadrantectomy	1 (2)
**Tumor size**	
1–2	21 (41.2)
3–5	26 (51)
> 5	4 (8.7)
**Involved lymph node metastasis**	
Yes	39 (76.47)
No	12 (23.52)
**Number of lymph nodes involved**	
< 2	43 (84.3)
≥ 2	8 (15.7)
**Nipple involvement**	
Yes	8 (15.7)
No	43 (84.3)
**Histologic grade**	
1/3	12 (23.5)
2/3	35 (68.6)
3/3	4 (8.7)
**Perivascular, neural invasion**	
Positive	21 (41.2)
Negative	30 (58.8)
**Taking anabolic drugs**	
Yes	5 (9.8)
No	46 (90.2)
**Family history of cancer**	
Yes	24 (47.05)
No	27 (54.90)
**Surgery and chemotherapy**	
Yes	43(84.3)
No	8 (15.7)
**Surgery and radiotherapy**	
Yes	27 (52.9)
No	24 (47.1)
**Surgery, radiotherapy, and chemotherapy**	
Yes	24 (47.1)
No	27 (52.9)
**Radiotherapy sessions**	
1–2	2 (11.74)
3–4	9 (52.94)
5–7	6 (35.29)
8–12	10 (5.88)

### Immunohistochemical characteristics

ER, PR, AR, Her2, P53, and Ki-67 were found to be positive in 38 cases (74.5%), 34 cases (66.7%), 20 cases (39.2%), 21 cases (41.17%), and 33 cases (64.7%), respectively (Table 3).

**Table 3:** Common biomarkers characteristics in breast cancer patients (*n* = 51)

Variable	Number (%)
**Estrogen receptor status**	
Positive	38 (74.5)
Negative	13 (25.5)
**Progesterone receptor status**	
Positive	34 (66.7)
Negative	17 (33.3)
**Androgen receptor status**	
Positive	20 (39.2)
Negative	31 (60.8)
**HER2 receptor**	
Positive	21 (41.17)
Negative	30 (58.83)
**P53**	
Positive	21 (41.2)
Negative	30 (58.8)
**Ki-67**	
Positive	33 (64.7)
Negative	18 (33.3)
Low	9 (17.6)
Intermediate	4 (12.1)
High	20 (60.6)

### Outcome

**Fig. 1:**
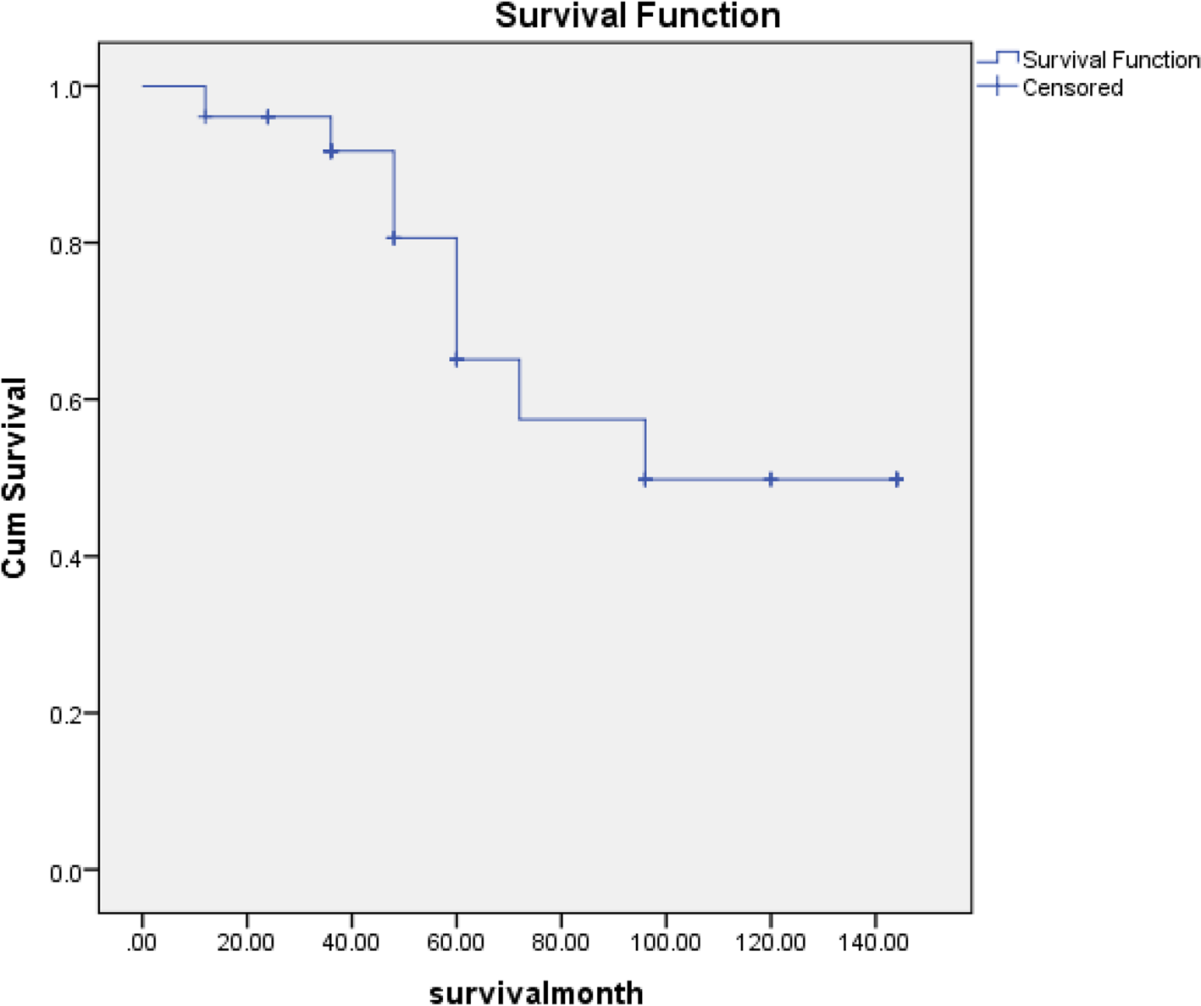
Overall survival for men with breast cancer

In this study, 17 cases (33.3%) of patients survived for more than 5 years, while 34 cases (66.7%) survived for less than 5 years. The overall survival rates at 1, 3, and 5 years for the cohort of 51 cases were 96%, 91%, and 65%, respectively, with an average survival duration of 96 months. Figure 1 illustrates the overall survival of breast cancer patients.

### The correlation between variables and patient survival rates

**Fig. 2:**
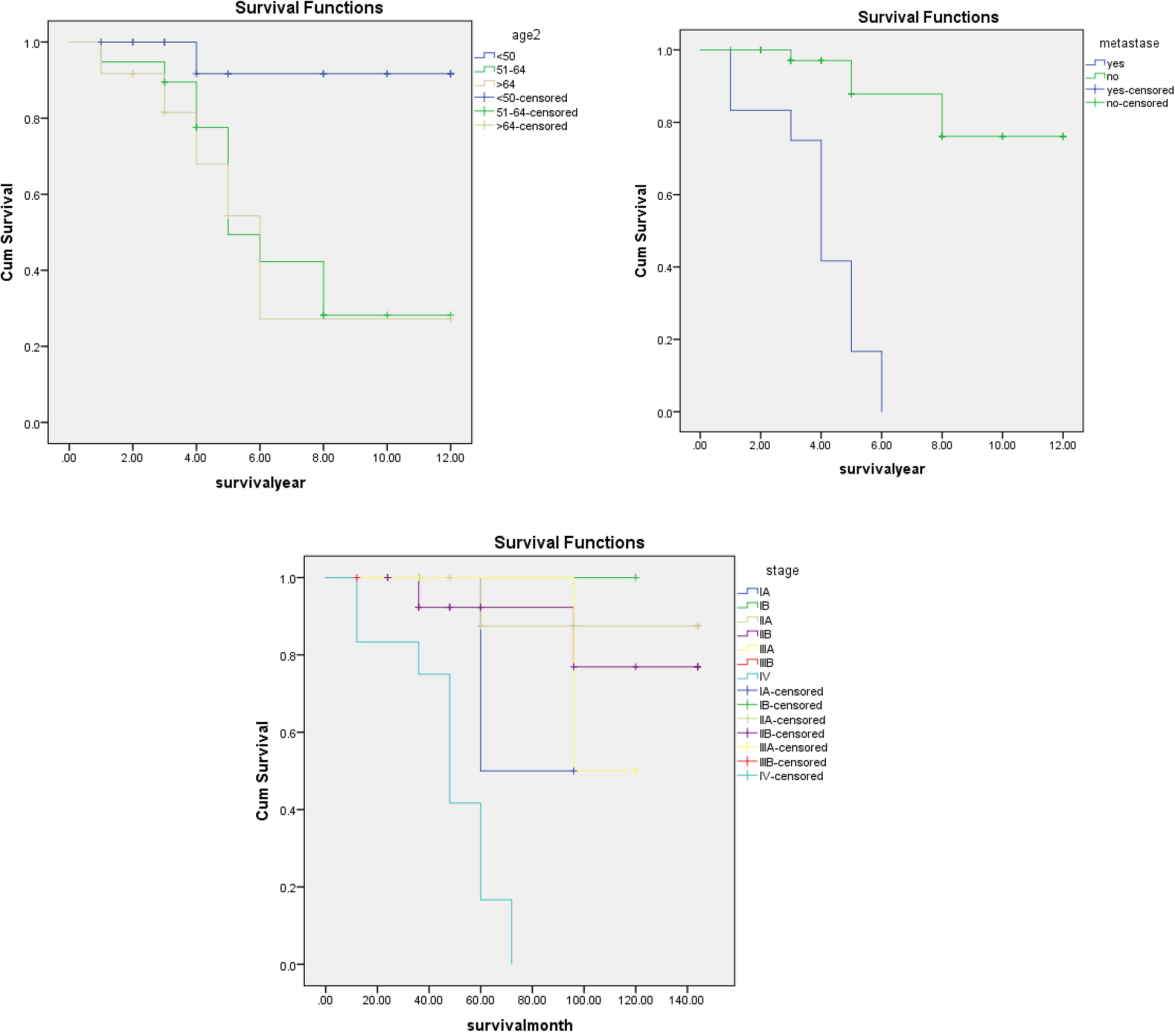
The survival of breast cancer patients based on the variables of age, metastasis, and disease stage

The data analysis indicates a significant correlation between age, metastasis, disease stage, and the survival status of patients (*P* = 0.03) (Fig. 2). Conversely, the survival rate of patients did not demonstrate a significant association with tumor location, tumor size, the involvement of lymph nodes, or receptor status.

### Correlation of HER2 variables with other variables

A positive and significant correlation was not observed between HER2 status and the variables of age, tumor type, metastasis, number of involved lymph nodes, estrogen and progesterone receptor status, P53 receptor status, use of anabolic drugs, family history, surgical intervention, and survival rate (*P* > 0.05).

## Discussion

Male breast cancer is a significant subject that has historically received less attention compared to female breast cancer. There has been some discourse regarding the prognostic factors and treatment strategies pertinent to male breast cancer. Over the past decade, the incidence of male breast cancer has been reported to have increased from 1 to 1.2 cases per 100,000 men between 1970 and 2004 [1].

In this study, the average age at which diagnosis occurred was 56 years, a figure that is lower than that reported in other studies. This discrepancy may reflect the differing age structures of various populations. Previous research has indicated that the average age at diagnosis of breast cancer in men ranges from 66 to 67 years, which is approximately 5 to 10 years older than the average age of diagnosis in women [2, 3].

The findings of one study indicated that the majority of breast cancers in men are classified as invasive ductal carcinomas. Papillary carcinoma is comparatively more prevalent, while lobular carcinoma is infrequently observed in the male population [1]. Ductal carcinoma in situ constitutes approximately 10% of breast cancer cases in men [14]. Furthermore, results from additional studies revealed that 84% of the identified cancer types were ductal, with mucinous and mixed forms following in prevalence [15–17]. Consistent with these previous studies, the current investigation found that 44 cases (86.3%) of the patients were diagnosed with invasive ductal carcinoma.

According to Giordano et al. [2], men aged 65 and older exhibit an increased risk of mortality associated with breast cancer. The study identified a statistically significant positive correlation between patient age and survival status. Men are often diagnosed at more advanced stages of the disease, potentially due to a lack of awareness regarding the characteristics of male breast cancer or the subtle differences and symptoms in the breast that necessitate medical evaluation. Additionally, in contrast to women, men do not undergo routine screening for breast cancer. Given the rarity of breast cancer in men, mammography is not recommended as a screening modality.

The majority of patients included in the study were diagnosed with stage 1, 2, 3, or 4 breast cancer. Lymph node metastasis was identified in 76.47% of the participants. This observation aligns with findings from previous research indicating that breast cancer in males is often diagnosed at more advanced stages compared to females, which may be attributed to delays in diagnosis [3, 6]. Mangone et al. reported that 39.5% of male patients were classified as stage 1, 33.1% as stage 2, 20.9% as stage 2, and 6.4% as stage 4 [16]. In the current study, the distribution of patients across the stages was as follows: 13.8% in stage 1, 54.9% in stage 2, 11.9% in stage 3, and 23.5% in stage 4. Furthermore, the overall survival rate for men diagnosed with breast cancer is lower than that for women, which can be attributed to factors such as older age and more advanced disease at the time of presentation and diagnosis [2].

In this study, we found that a higher stage at diagnosis was correlated with an increased risk of mortality. Conversely, no statistically significant association was identified between lymph node involvement and the survival status of patients. This lack of correlation may be attributed to the low prevalence of breast cancer in men and the limited sample size. Furthermore, our findings indicate that more than two-thirds of the patients underwent mastectomy, which aligns with previous reports [4, 5]. This contrasts with female breast cancer, where approximately two-thirds of patients receive breast-conserving surgery, while one-third undergo mastectomy. The disparity in the rates of breast-conserving surgery versus mastectomy among overweight men may be influenced by concerns regarding the adequacy of surgical margins, as the small size of male breasts may hinder the complete removal of at-risk breast tissue. Additionally, differences in aesthetic considerations between genders may also play a role. It is noteworthy that male breast cancers are frequently centrally located and involve the nipple, necessitating the excision of the nipple-areolar complex [18].

Male breast cancer exhibits a significant prevalence of receptor positivity for estrogen and progesterone [6]. In our investigation, we observed that among 22 male patients diagnosed with cancer, 74.5% were found to be estrogen receptor (ER) positive, while 66.7% were progesterone receptor (PR) positive. Previous studies have reported positive estrogen receptor rates of 96.4%, 94%, and 84%, which are notably higher than the findings of our study [16, 17, 19]. Additionally, the prevalence of positive progesterone receptors in other research has ranged from 86 to 88%, again surpassing the percentage observed in our study [20, 21]. Furthermore, Ki-67 levels below 10% were identified in 64.7% of the patients, which is higher than the figures reported by Mangone et al. [16] and Silvestri et al. [21] yet lower than the 75.3% reported in Cardoso’s study [15]. In terms of HER2 overexpression, previous studies have indicated a range from 2 to 42% [7, 8], whereas our study found HER2 overexpression to be 41.17%.

Chemotherapy is recommended as adjuvant therapy for younger patients presenting with larger tumors and axillary lymph node involvement. In our study, adjuvant chemotherapy was administered to 84.3% of patients with larger tumors and/or lymph node involvement, which encompassed 62.8% of individuals classified as stage 2 or 3 and 76.47% of those who were node positive.

Overall survival rates are lower for men, which can be attributed to their older age at diagnosis and the more advanced stage of disease at presentation [2]. Specifically, the 3-year survival rate for men is 86.4%, while the 5-year survival rate is 77.6% [22]. In contrast, this study reported the 3-year and 5-year survival rates as 91% and 65%, respectively.

This study acknowledges certain limitations, one of which pertains to its retrospective design involving a relatively small cohort of patients treated over a span of 10 years. This limitation is largely attributable to the rarity of male breast cancer, which poses challenges in accumulating a substantial patient population from a single institution.

## Conclusions

Considering the relationship between age and disease survival suggests that the measures required for the early detection of male breast cancer are comparably effective to those for female breast cancer. Given the rarity of male breast cancer, it is imperative to conduct larger studies that involve collaboration among multiple research centers specializing in breast cancer. In our investigation, we found that older age, advanced stage, and the presence of metastasis in male breast cancer were associated with poorer survival outcomes, consistent with findings from previous studies. Furthermore, the overall survival rate observed in our study aligns with the results reported in the existing literature.

## Data Availability

Data is provided within the manuscript.
